# Assessing patterns in cancer screening use by race and ethnicity during the coronavirus pandemic using electronic health record data

**DOI:** 10.1002/cam4.6246

**Published:** 2023-06-22

**Authors:** Fredric E. Blavin, Laura Barrie Smith, Lisa Dubay, Luis Basurto

**Affiliations:** ^1^ Health Policy Center Urban Institute Washington District of Columbia USA; ^2^ Duke University, Sanford School of Public Policy Durham North Carolina USA

**Keywords:** cancer screening, colonoscopies, COVID‐19, EHR data, mammograms, racial and ethnic disparities

## Abstract

**Background:**

Efforts to prevent the spread of the coronavirus led to dramatic reductions in nonemergency medical care services during the first several months of the COVID‐19 pandemic. Delayed or missed screenings can lead to more advanced stage cancer diagnoses with potentially worse health outcomes and exacerbate preexisting racial and ethnic disparities. The objective of this analysis was to examine how the pandemic affected rates of breast and colorectal cancer screenings by race and ethnicity.

**Methods:**

We analyzed panels of providers that placed orders in 2019–2020 for mammogram and colonoscopy cancer screenings using electronic health record (EHR) data. We used a difference‐in‐differences design to examine the extent to which changes in provider‐level mammogram and colonoscopy orders declined over the first year of the pandemic and whether these changes differed across race and ethnicity groups.

**Results:**

We found considerable declines in both types of screenings from March through May 2020, relative to the same months in 2019, for all racial and ethnic groups. Some rebound in screenings occurred in June through December 2020, particularly among White and Black patients; however, use among other groups was still lower than expected.

**Conclusions:**

This research suggests that many patients experienced missed or delayed screenings during the first few months of the pandemic, which could lead to detrimental health outcomes. Our findings also underscore the importance of having high‐quality data on race and ethnicity to document and understand racial and ethnic disparities in access to care.

## 1 INTRODUCTION

Efforts to prevent the spread of the novel coronavirus led to historic reductions in the volume of nonemergency medical care services provided during the first several months of the COVID‐19 pandemic. While disruptions to in‐person care were partially offset by a rapid increase in telehealth services,^1^, ^2^, ^3^, ^4^ some health care procedures, including many types of cancer screenings, could not be provided via telehealth. A dramatic decline in breast cancer screenings during the first several months of the pandemic among commercially insured individuals has been documented,^5^ and evidence suggests similar patterns occurred for other preventive health care services (e.g., cervical and colorectal cancer screenings) as well.^6^, ^7^ Delayed or missed screenings can lead to more advanced stage cancer diagnoses with potentially worse health outcomes as a result.^8^

The pandemic brought to the public's attention long‐standing health care disparities. Systemic racial and ethnic inequities pervade the U.S. health care system, and cancer‐related care is no exception. Black, Indigenous, and people of color (BIPOC) individuals are more likely than non‐Hispanic White individuals to receive advanced‐stage breast cancer diagnoses, contributing to higher rates of breast cancer morbidity and mortality.^9^, ^10^, ^11^, ^12^ There are also significant racial and ethnic disparities in colorectal screenings and mortality rates, especially for Black individuals.^11^, ^12^, ^13^, ^14^

It is not known to what extent cancer screening volume changed differentially for patients of different races and ethnicities during the first 10 months of the COVID‐19 pandemic in the United States. However, evidence from early in the pandemic indicates that Black non‐elderly adults were more likely to forgo or delay needed care compared to White and Hispanic non‐elderly adults due to both fear of contracting COVID and their health care providers remaining closed.^15^ Communities of color may also have been disproportionately impacted by public transportation disruptions, creating additional access problems.^16^ Several studies documented declines in cancer screenings during the first few months of the pandemic.^5^, ^17^, ^18^, ^19^, ^20^, ^21^, ^22^ While these studies suggested some differences across racial and ethnic groups, the literature is generally limited by region of study, sample size, or lack of available information on the racial and ethnic composition of those getting cancer screenings.

In this study, we used novel data from a national electronic health record (EHR) vendor to assess how the volume of breast and colorectal cancer screenings (i.e., mammograms and colonoscopies) changed before and after the pandemic started, from 2019 through 2020. We also contribute to the literature by testing whether there were significant differences in these changes by race and ethnicity. Combined, we contribute to the literature by examining changes in breast and colon cancer screening—overall, within, and across racial and ethnic groups—over the first 10 months of the pandemic among a large U.S. adult population.

## 2 METHODS

### 2.1 Data and study sample

We used data from the COVID‐19 Research Database, a public–private consortium organized to enable the use of real‐world data to better understand the COVID‐19 pandemic. Specifically, we used a national sample of de‐identified ambulatory care EHR data from Veradigm (formerly Allscripts), a large health information, analytics, and intervention solutions company that provides EHR and practice management products to physician practices nationwide and reaches over 58 million patients. Key fields used from the Veradigm EHR data include patient demographics (age, race and ethnicity, gender, and state of residence), provider characteristics (type, title, and specialty) encounter and order type, and procedure codes.

We analyzed data on two samples of providers that placed orders in 2019–2020 for mammogram screenings for breast cancer and colonoscopy screenings for colorectal cancer. We first identified these cancer screening services using the Healthcare Common Procedure Coding System (HCPCS) procedure codes. We excluded orders that were canceled and orders that were duplicated for a given patient on a given day. Next, for each sample, we included a panel of providers that ordered the screening in any month of the study period. However, to ensure we had a consistent panel of providers, we restricted the sample to providers who had at least 30 patient encounters (of any type, not limited to cancer screenings) each month of 2019–2020, excluding March–May 2020 when in‐person services were largely unavailable for some types of providers. This sample restriction excludes providers that dropped out of the Veradigm EHR data altogether (e.g., retirees) or entered after the start of the analysis period (e.g., new practices). Both samples include orders from all 50 states plus the District of Columbia. Compared to the population of US adults ages 50–74, the patients seen by the provider sample overrepresent the South and underrepresent the Midwest (Appendix Table S1).

We also excluded orders from July 2020 due to unexplained reporting gaps in the Veradigm EHR data during this month only. We therefore excluded July 2019 data in instances when we make monthly year‐to‐year comparisons (i.e., Figures 3 and 4). See appendix for additional details on the sample creation process.

To examine differences by race and ethnicity, we used the patient race and ethnicity information recorded on patients' demographic records included as variables in the dataset. The race categories provided in the data included White, Black or African American, Asian, “other”, and unknown or null. The ethnicity categories included Hispanic or Latino and not Hispanic or Latino. We used the race and ethnicity variables to create a combined classification for each patient. Specifically, we classified any patients with an ethnicity of Hispanic or Latino as “Hispanic or Latino” regardless of their race, and all other patients were classified according to their reported race.

### 2.2 Data quality

One challenge of this study is the extent of missing data in the race and ethnicity data fields. We analyzed the distribution of mammograms and colonoscopies by patient race and ethnicity over time to assess the quality (i.e., “missingness”) of the race and ethnicity data. Appendix Figure S1 shows the share of mammogram orders associated with unknown race and ethnicity data slightly declined over the analysis period. Overall, the share of mammogram orders associated with unknown race and ethnicity declined from 14.2% in the pre‐pandemic period (January 2019–February 2020) to 13.0% in the post‐pandemic period (March 2020–December 2020) (Appendix Table S2).

Colonoscopies were associated with more monthly fluctuations and higher rates of unknown race and ethnicity data (Appendix Figure S2). However, these unknown rates decreased over time, from 27.2% of all colonoscopies in the pre‐pandemic period to 23.7% in the post‐pandemic period (Appendix Table S2).

### 2.3 Statistical analyses

For each cancer screening outcome, we tallied the total number of services ordered for each month of 2019 and 2020 for each provider. We then calculated the weighted average number of services per month per provider for all patients and for each race and ethnicity category. We then examined how the weighted average number of services for each month differed between 2019 and 2020 for all patients.

We used a difference‐in‐differences (DiD) analysis to examine the extent to which changes in provider‐level mammogram and colonoscopy orders were reduced over the first 10 months of the pandemic (i.e., March 2020–December 2020), relative to the same months in 2019 to control for time‐variant factors, and whether these changes were significantly different across race and ethnicity groups. Traditional DiD uses data from treatment groups and control groups to obtain a counterfactual to estimate a causal effect over time. In our analysis, we do not have a treatment and control group per se; instead, for each month, we estimated the change in weighted average mammograms and screenings from March 2019–December 2019 (“pre‐period”) to March 2020–December 2020 (“post‐period”) for each non‐White race and ethnicity group relative to outcomes for White, non‐Hispanic patients.

For each month except for July, we estimated fixed‐effect OLS models, where the outcome is the number of orders, separately for each panel of providers in the mammograms and colonoscopy sample. We applied the significance tests from these models to determine if the percentage change in screenings significantly differed across race and ethnicity groups. These standard errors are an upper‐bound approximation of the true standard errors of the percentage change in levels.^23^ We focus on the percentage change in levels because the absolute changes are less meaningful as the White group constitutes the largest number of patients, and we could not use log transformed models due to the significant number of observations with 0 values.

As an alternative to month‐specific models, we also estimated models separately for the initial phase of the pandemic (Period 1, March 2020–May 2020 treatment exposure compared to March 2019–May 2019) and the later phase when service use picked up (Period 2, June 2020–December 2020 treatment exposure compared to June 2019–December 2019). We also estimated models that consider the whole post‐pandemic phase (March 2020–December 2020 treatment exposure compared to March 2019–December 2019).

## 3 RESULTS

### 3.1 Provider characteristics

Our sample includes 5590 providers that ordered mammograms and 3077 providers that ordered colonoscopies from 2019 through 2020 (Table 1). Providers for both procedures varied by provider type and specialty as can be seen in Table 1. Overall, the weighted average number of mammograms per provider per month fell from 12.3 in March 2019–December 2019 to 11.0 in March 2020–December 2020, a relative decline of 10.6%. The average number of colonoscopies per provider per month fell from 8.8 to 7.7 during this same period, a relative decline of 12.5%.

**TABLE 1:** Descriptive characteristics of providers, by sample.

	Mammograms	Colonoscopies
Total number of providers	5590	3077
Average number of orders per month, Jan 2019–Feb 2020 (provider‐level)	12.3	8.8
Average number of orders per month, Mar 2020–Dec 2020 (provider‐level)	11.0	7.7
*Provider type (%)*		
Allopathic and Osteopathic Physicians	69.8	70.7
Physician assistants and advanced Practitioners	24.0	23.1
Other	1.9	1.7
Missing	4.3	4.6
*Specialty (%)*		
Primary care	69.0	71.3
ObGyn/Midwife/WHNP (mammograms) or Gastro/surgical (colonoscopies)	9.6	15.1
Other specialty	17.1	9.0
Missing	4.3	4.6

*Source*: Authors' analysis of Veradigm ambulatory care electronic health record data from the COVID‐19 Research Database.

### 3.2 Changes in mammograms and colonoscopies amid COVID‐19

**FIGURE 1:**
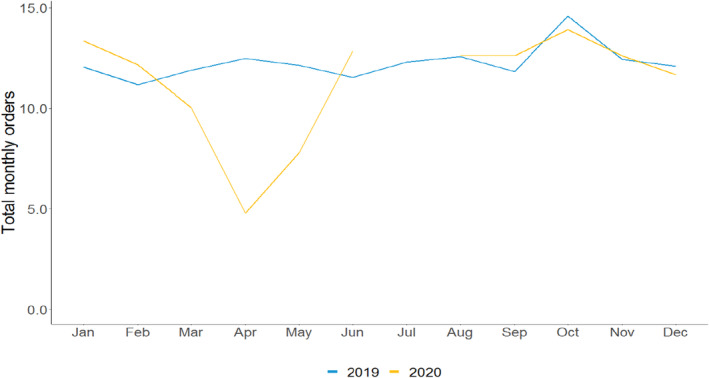
Average number of mammograms per provider, by month. *Source*: Authors' analysis of Veradigm ambulatory care electronic health record data as part of the COVID‐19 Research Database.

**FIGURE 2:**
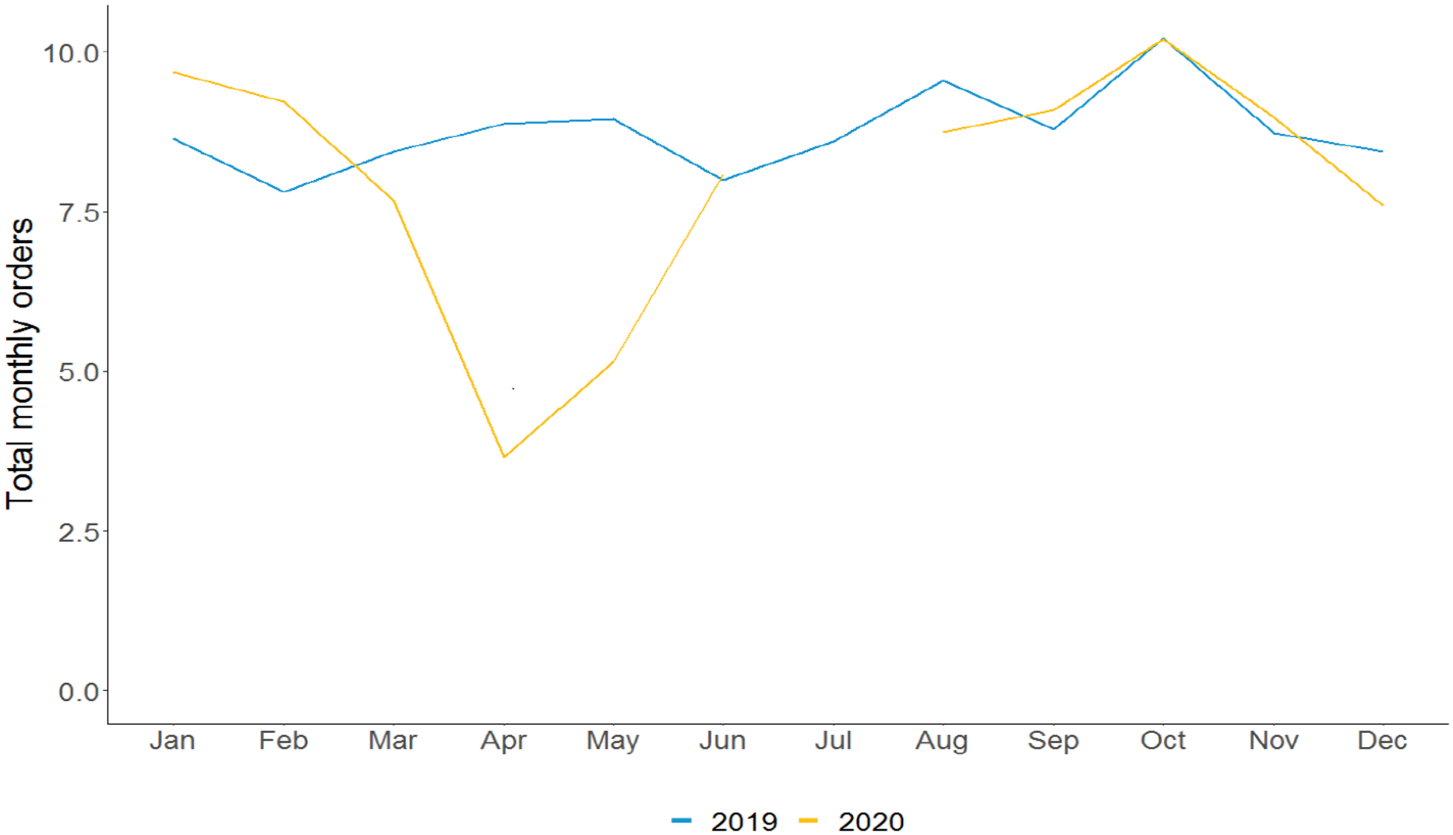
Average number of colonoscopies per provider, by month. *Source*: Authors' analysis of Veradigm ambulatory care electronic health record data as part of the COVID‐19 Research Database.

We first assess how orders for mammograms (Figure 1) and colonoscopies (Figure 2) changed during the first 9 months of the pandemic by comparing the average number of orders per provider per month in 2019 and 2020. The number of mammograms and colonoscopies per provider in January and February 2020—just prior to the onset of the pandemic—were somewhat higher than levels in January and February 2019. However, in a reversal, mammograms per provider fell from 11.9 in March 2019 to 10.0 in March 2020, a relative decline of 16%. Mammograms per provider fell further in April, declining from 12.5 in April 2019 to 4.8 in April 2020, a relative decline of 62%. The pattern was similar for colonoscopies per provider, which dropped from 8.6 to 7.8 in March 2019 compared to March 2020 and from 9.1 to 3.7 in April 2019 and April 2020, respectively. These reductions in procedures represent relative declines of 9% and 59%, respectively. Use of both procedures picked up in May 2020 (after most outpatient practices resumed providing in‐person care^24^) and reached 2019 levels in June 2020. The levels and patterns of care from August through December 2020 were like those observed in August through December 2019.

### 3.3 Changes in mammograms and colonoscopies by race amid COVID‐19

**FIGURE 3:**
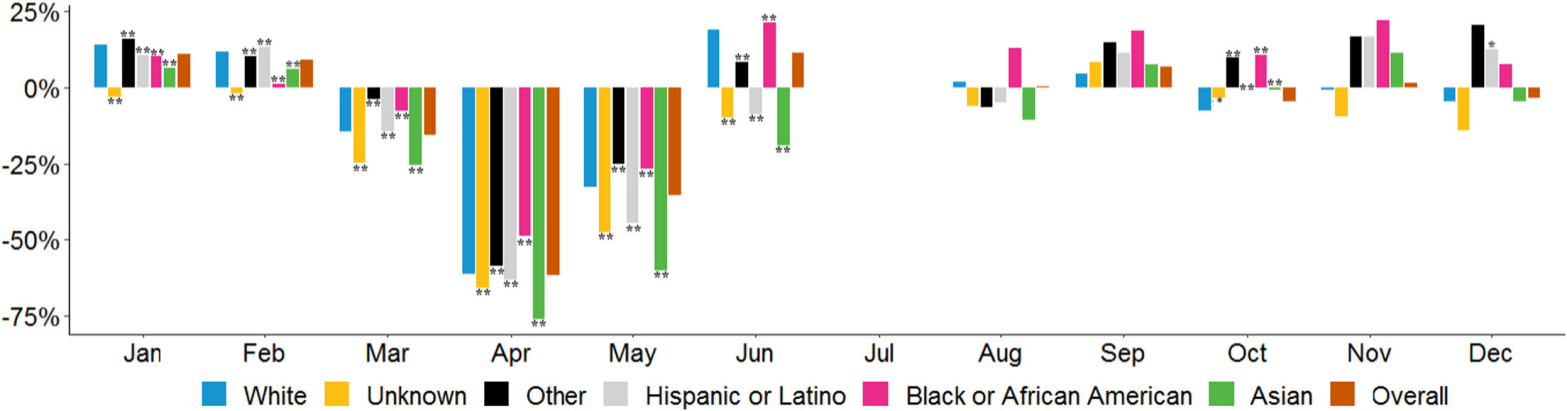
Percentage change in year‐to‐year mammograms, by race/ethnicity and month. *Source*: Authors' analysis of Veradigm ambulatory care electronic health record data as part of the COVID‐19 Research Database. The reference group in each year is non‐Hispanic Whites. **/* Denotes statistical significance at the 1%/5% levels.

**FIGURE 4:**
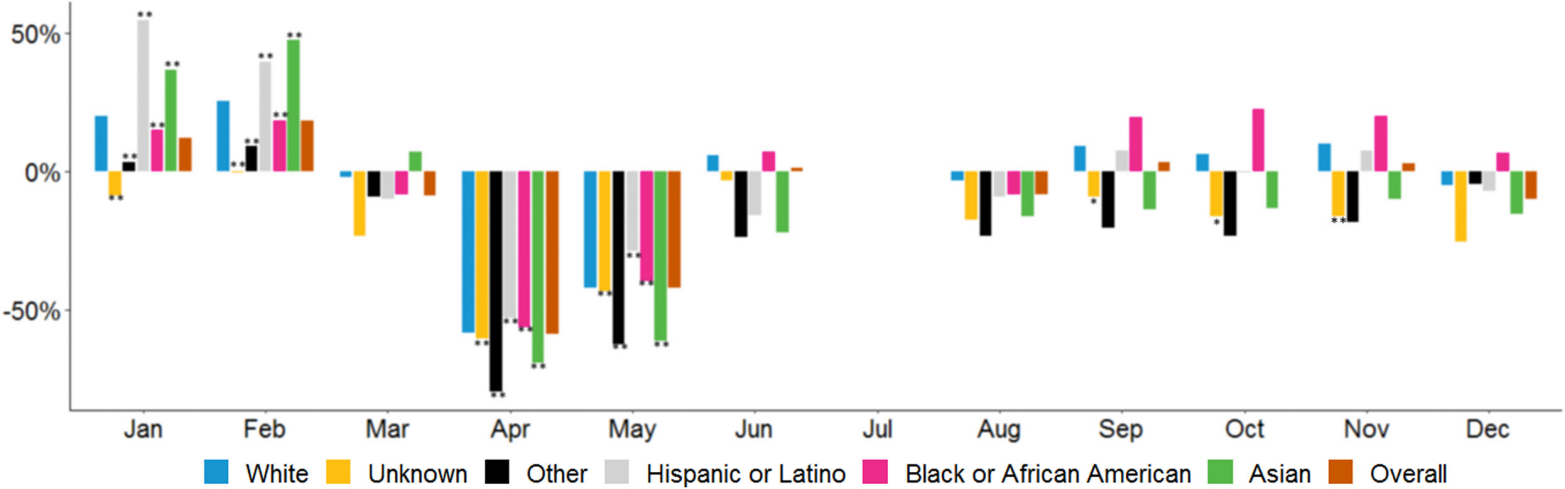
Percentage change in year‐to‐year colonoscopies, by race/ethnicity and month. *Source*: Authors' analysis of Veradigm ambulatory care electronic health record data as part of the COVID‐19 Research Database. The reference group in each year is non‐Hispanic Whites. **/* Denotes statistical significance at the 1%/5% level.

Figure 3 shows the monthly percentage change in mammograms per provider between 2019 and 2020 and Figure 4 shows the monthly changes in colonoscopies per provider by race and ethnicity groups. Leading up to the pandemic, both mammograms and colonoscopies increased in January and February 2020 relative to 2019 for nearly all racial and ethnic groups. In a reversal of these trends, preventive service use declined for all groups in the early phase of the pandemic (March 2020–May 2020)—with the largest declines occurring in April 2020—while utilization started to rebound in subsequent months (June–December 2020).

For mammograms, the percentage declines in March, April, and May 2020 (relative to March, April, and May 2019, respectively) were largest for Asian patients, patients whose race is unknown, and Hispanic or Latino patients, while in general, the percentage declines were smaller for White and Black or African American patients. Estimates from the DiD model imply that changes in screenings for White patients differed significantly from the changes for all other racial and ethnic groups during the early phase of the pandemic with Black or African American and “other” race patients having smaller reductions in use than White patients. In contrast, in the later part of the pandemic (June 2020–December 2020 or the “rebound” period), there were fewer statistically significant differences in mammogram screenings across racial and ethnic groups, and most of these differences were concentrated in June and October. For example, White and Black or African American patients experienced larger percentage increases in mammograms in June, while other racial and ethnic groups experienced a delayed rebound effect in subsequent months (e.g., the decline in mammograms among White patients in October significantly differed from the changes among all other racial and ethnic groups). Mammograms increased in every month in this period for Black or African American patients (relative to 2019), the only racial and ethnic group to experience this pattern.

There were also declines in colonoscopies across all racial and ethnic groups in April 2020–May 2020 with some rebound in later months, but the patterns were not nearly as consistent or uniform as those seen for mammograms. Estimates from the DiD model imply that the change in screenings for White patients differed from the changes for all other racial and ethnic groups during April and May 2020 relative to April and May 2019. However, the rebound effect was generally small or nonexistent in June and August (e.g., colonoscopies declined relative to 2019 levels) for all racial and ethnic groups. In fact, for Asian patients and patients of “other” racial and ethnic groups, colonoscopy levels were lower in each month in June–December 2020 relative to 2019. Overall, from June through December 2020, there were no significant differences in colonoscopies between White patients and all other racial and ethnic groups (except for the unknown category).

Table 2 highlights the relative percentage changes in mammograms and colonoscopies for each racial and ethnic group during the initial phase of the pandemic (Period 1, March 2020–May 2020 relative to March 2019–May 2019), the later phase when service use picked up (Period 2, June 2020–December 2020 relative to June 2019–December 2019), and the whole post‐pandemic phase. The table also indicates where the DiD estimates are statistically significant, that is, where the difference in the percentage change for White patients differ for all other groups of patients.

**TABLE 2:** Percentage change in mammograms and colonoscopies per provider from 2019 to 2020, by race and ethnicity overall and by pandemic period.

	Period 1 Mar–May 2020 vs. Mar–May 2019		Period 2 June–Dec 2020 vs. June–Dec 2019		Overall Mar–Dec 2020 vs. Mar–Dec 2019	
*Mammograms*						
Asian	−54%	***	−3%		−20%	***
Black or African American	−28%	***	15%		0.4%	***
Hispanic or Latino	−41%	***	4%		−11%	***
Other	−30%	***	11%		−2%	***
Unknown	−47%	***	−6%	**	−19%	***
White	−36%		2%		−11%	
All race and ethnicity groups	−38%		2%		−11%	
*Colonoscopies*						
Asian	−44%	***	−15%	*	−23%	***
Black or African American	−35%	***	11%		−5%	***
Hispanic or Latino	−30%	***	−3%	*	−12%	***
Other	−52%	***	−19%	*	−30%	***
Unknown	−43%	***	−15%	***	−24%	
White	−35%		4%		−9%	
All race and ethnicity groups	−37%		−2%		−13%	

*Source*: Authors' analysis of Veradigm ambulatory care electronic health record data from the COVID‐19 Research Database.

***/**/* Denotes statistical significance at the 1%/5% levels/10% levels of the difference in the percentage change for White patients versus all other groups of patients.

Findings from the period‐specific models are consistent with the differences observed with the month‐to‐month percentage changes observed in Figures 3 and 4. For example, in period 1 (March through May), the changes in mammograms and colonoscopies statistically differed for patients in each of the race and ethnicity groups, relative to the declines in orders for White patients. For example, for mammograms, relative to White patients, the percentage declines were larger for those in Asian, Hispanic/Latino, and unknown race and ethnicity groups, and the percentage declines were smaller for Black/African American and “other” race and ethnicity populations. In contrast, in period 2, across both the mammogram and colonoscopy models, there were fewer statistically significant differences across racial and ethnic groups, and most of these differences are concentrated in the colonoscopy model.

## 4 DISCUSSION

The COVID‐19 pandemic temporarily halted nearly all nonurgent medical care services. Using a large EHR dataset, we found that screenings for mammograms and colonoscopies significantly declined in March through May 2020 relative to their monthly levels in 2019; in fact, in April 2020, screenings for mammograms and colonoscopies declined by over 50% of their 2019 levels, a finding that is consistent with prior studies.^25^, ^26^, ^27^ Service use eventually began to rebound, as the overall level of screenings from June through December 2020 was like those observed in June through December 2019. This is consistent with previous studies.^5^, ^6^, ^7^ Overall, missed screenings were not made up for by the end of 2020 when the pandemic was still early in its evolution and vaccines were not yet available for most people.

We also found significant differences in the changes in screenings across racial and ethnic groups. During the early phase of the pandemic, there were large declines in mammograms for all racial and ethnic groups, most notably among the Asian population. Some rebound in screenings occurred in June through December 2020, particularly among White and Black or African American patients; however, use among other groups was still lower than it had been during this period in 2019.

These findings are important because missed screenings could potentially delay diagnoses and treatment for cancer, especially for high‐risk individuals. For example, for some patients, a missed or delayed mammogram could lead to a larger tumor or a breast cancer diagnosis at a more advanced stage.^8^, ^28^ Similarly, delays in undergoing colonoscopy following an abnormal stool test can increase the risk of a colorectal cancer diagnosis and cancer‐related death.^29^, ^30^ In contrast, however, for low‐risk women, some delay in mammograms or colonoscopies may cause no harm.^30^, ^31^ These findings highlight the importance of follow‐up care following delayed screenings for those with elevated risks for breast or colorectal cancer. A national quality improvement study, Return‐to‐Screening, was effective at increasing screening at cancer centers to pre‐pandemic levels through a variety of evidence‐based interventions designed to increase patient demand, screening delivery, and community services. The Return‐to‐Screening study did not examine impacts by the race and ethnicity of patients; however, the results show that cancer screenings can be increased using a combination of quality improvement strategies.^32^

Our approach and the EHR dataset have several limitations. While our approach accounts for the seasonality of mammograms and colonoscopies, it does not take into account the potential for annual or month‐to‐month trends in the procedures prior to the pandemic which, if present, would bias our estimated effects. However, we find that the overall trends leading up to COVID‐19, overall and by race and ethnicity, were relatively constant or slightly upwards, which suggests that if anything, our findings provide a lower bound estimate of the pandemic's impact. This result is consistent with prior research showing that trends in mammograms and colonoscopies were constant leading up to and during 2019.^33^ In addition, the EHR dataset is not nationally representative and it does not include measures that would help us assess health impacts or patients' risk for cancer. There are also significant missing data on race and ethnicity. The quality of race and ethnicity reporting in the data significantly varies across mammograms and colonoscopies. High rates of unknown race and ethnicity associated with colonoscopies could be due to differences in patient characteristics (e.g., potential gender differences in the likelihood of self‐reporting race and ethnicity) or differences in provider characteristics (e.g., gastroenterologists could be less likely to record race and ethnicity data than primary care providers). Even though the reporting of patient race and ethnicity in our sample of EHR data improved over time, there is still a need for better data on race and ethnicity—a challenge that is not limited to EHR databases.^34^

As the COVID‐19 pandemic continues to evolve, continued research is needed to understand if patterns in cancer screenings have changed since 2020 and whether there are persistent differences in access to care more broadly by race and ethnicity. A better understanding of the reasons for the variation in patterns across racial and ethnic groups is needed, including whether access to providers, fear of COVID, language and cultural barriers, and/or other factors played a role in the differential impact on screening to inform providers serving patients in their communities. For example, restrictions on patients' ability to have someone accompany them to health care visits may have made it more difficult for those with language issues to access services.^35^ Each of these challenges could reappear depending on the transmission and virulence of future waves of the pandemic. Future research should also evaluate the health impacts of missed or delayed screenings, with a focus on racial and ethnic inequities.

## AUTHOR CONTRIBUTIONS

**Fredric Blavin:** Conceptualization (equal); data curation (equal); formal analysis (equal); investigation (equal); methodology (equal); project administration (supporting); supervision (equal); validation (supporting); visualization (supporting); writing – original draft (lead); writing – review and editing (lead). **Laura Barrie Smith:** Conceptualization (equal); data curation (equal); formal analysis (equal); methodology (equal); supervision (supporting); validation (supporting); visualization (supporting); writing – review and editing (supporting). **Lisa Dubay:** Conceptualization (lead); data curation (supporting); formal analysis (supporting); funding acquisition (lead); investigation (equal); methodology (supporting); project administration (lead); resources (lead); software (supporting); supervision (lead); validation (supporting); visualization (supporting); writing – original draft (supporting); writing – review and editing (supporting). **Luis Basurto:** Data curation (equal); formal analysis (supporting); methodology (supporting); software (lead); validation (equal); visualization (lead).

## FUNDING INFORMATION

This project was funded by a grant from the Merck Foundation.

## CONFLICT OF INTEREST STATEMENT

No potential conflicts of interests exist.

## ETHICAL COMMITTEE/INSTITUTIONAL REVIEW BOARD STATEMENT

This study was exempt from the Urban Institute's Institutional Review Board (IRB) since this research did not involve human subjects or personally identifiable information.

## Supporting information


Appendix S1.

## Data Availability

The data, technology, and services used in the generation of these research findings were generously supplied pro bono by the COVID‐19 Research Database partners, who are acknowledged at https://covid19researchdatabase.org/. Restrictions apply to the availability of these data, which were used under license for this study.
