# Supplementary material for: Assessing patterns in cancer screening use by race and ethnicity during the coronavirus pandemic using electronic health record data
Source: Cancer Med. 2023 Jun 22;12(15):16548–57. doi: 10.1002/cam4.6246 (PMC10469733; doi:10.1002/cam4.6246)
Supplement: Supplementary file 1 — Appendix S1. [file CAM4-12-16548-s001.docx]

**APPENDIX**

**Data**

Sample creation for mammograms and colonoscopies started by generating a variable to count the months a provider is represented in the data, then filtering for relevant CPT codes for each procedure. We also filter out orders that are marked as “canceled”, “entered in error”, “erroneous”, “voided”, “temporary deferral”, “permanent deferral”, “hold for”, “need information”, “unauthorized”, and “discontinued”. After filtering, there were still cases where multiple orders associated with the same order ID. Many of these orders were inconsistently labeled, making it difficult to know if orders were completed or in progress. To ensure we have one order for each order ID, we choose the most recent order for the listed ID. We also excluded orders from 2021, orders with missing provider ID’s, and orders with associated with providers who do not have a consistent panel of data.

This results in an order-level dataset of providers by month, year, and race. We sum the orders by providers to generate a provider-level dataset of orders by month, year, and race. To ensure we have the same number of providers in both specified periods (Jan 2019-Feb 2020 and March 2020-Dec 2020), we expanded the order level dataset by adding empty observations so that the total observation value equals the unique providers multiplied by six (to represent the race categories) and 23 (to represent all of the months in the sample).

To assess the national representativeness of the resulting samples, we compared the regional distribution of patients in the mammogram and colonoscopy sample to the national population of adults ages 50-74, and the results are shown in Table S1 below.

Table S1: Regional Representativeness of Analytic Samples

|  | Share of Individuals | | |
| --- | --- | --- | --- |
| Census Region: | National (%) | Veradigm Mammograms Sample (%) | Veradigm Colonoscopies Sample (%) |
| Midwest | 20.7 | 13.0 | 12.4 |
| Northeast | 17.2 | 14.2 | 19.6 |
| South | 38.4 | 47.4 | 44.0 |
| West | 23.7 | 25.4 | 24.0 |
| *Total* | *100* | *100* | *100* |

NOTE: National figures based on population estimates from the U.S. Census Bureau for adults ages 50-74 in 2020. Veradigm estimates are based on the distribution of patients seen by the provide samples.

**Multivariate findings (absolute changes in orders)**

While the estimates in the figures represent percent changes, the DiD estimates (data not shown) represent the relative change in use, from 2019 to 2020, for each race and ethnicity group relative to the change in use for white patients. These estimates provide information on whether the declines for each race/ethnicity group are statistically different from one another, with non-Hispanic white patients being the reference group.

Overall, DiD estimates indicate that the *absolute* decline in services was larger for non-Hispanic white patients relative to all other racial and ethnic groups during the early phase of the pandemic. These differences were statistically different from one another in March-May for mammograms and only April-May for colonoscopies.

In contrast, in the later part of the pandemic (June-December 2020), there were fewer statistically significant differences across racial and ethnic groups, and most of these differences were concentrated in June and October in the mammogram model i.e., the increases in mammograms were larger for non-Hispanic white patients in June and smaller in October relative to all other racial and ethnic groups.

**Appendix Figure S1: Distribution of Race/Ethnicity for Mammograms, by Month**


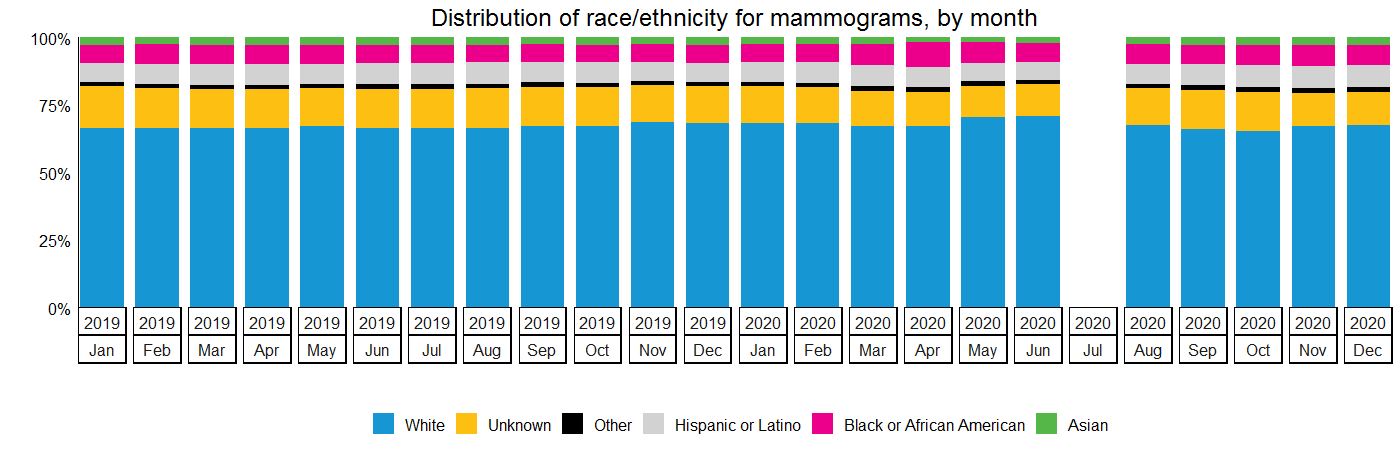


SOURCE: Authors’ analysis of Veradigm ambulatory care electronic health record data from the COVID-19 Research Database.

**Appendix Figure S2: Distribution of Race/Ethnicity for Colonoscopies, by Month**
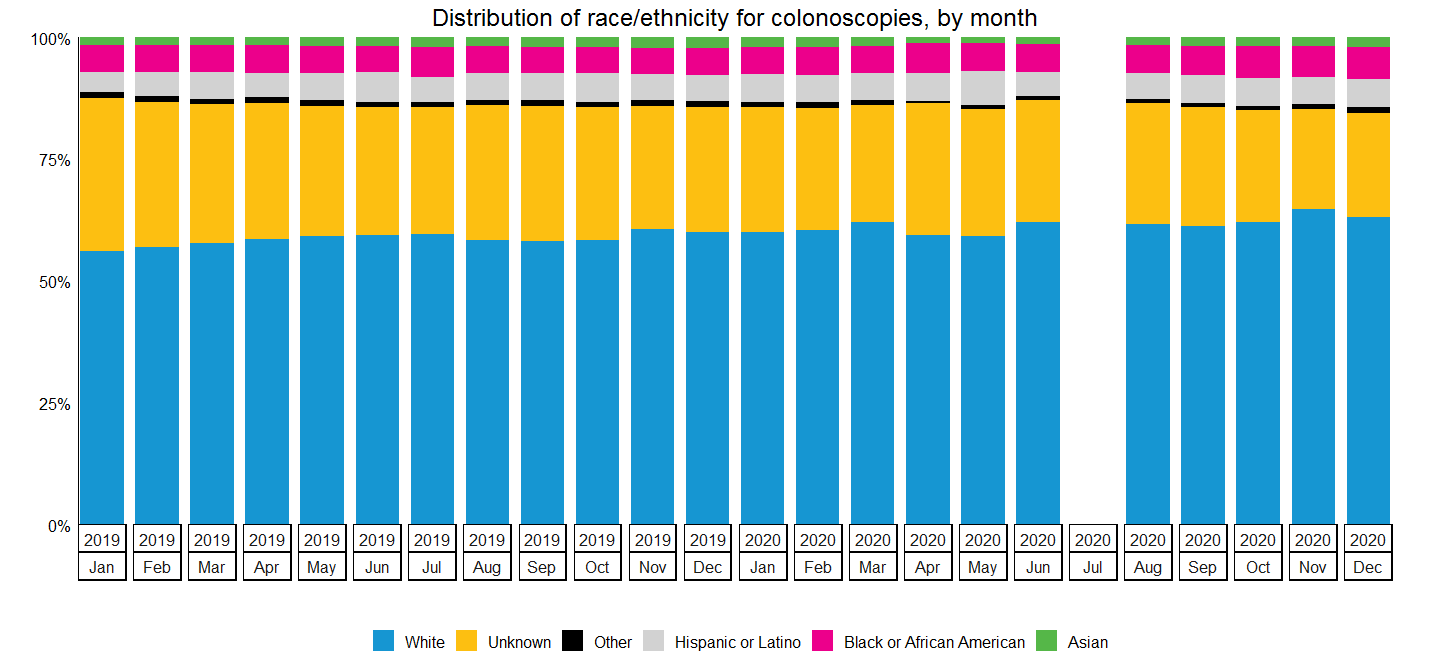


SOURCE: Authors’ analysis of Veradigm ambulatory care electronic health record data from the COVID-19 Research Database.

| **Appendix Table S2. Distribution of Mammograms and Colonoscopies by Race and Ethnicity and Age, Before (January 2019-February 2020) and After (March 2020-December 2020) the COVID-19 Pandemic Began** | | | | | | | | | | |
| --- | --- | --- | --- | --- | --- | --- | --- | --- | --- | --- |
|  |  | **Mammograms** | | | |  | **Colonoscopies** | | | |
|  |  | **Jan 2019-Feb 2020** | | **Mar 2020-Dec 2020** | |  | **Jan 2019-Feb 2020** | | **Mar 2020-Dec 2020** | |
|  |  | **N** | **%** | **N** | **%** |  | **N** | **%** | **N** | **%** |
| **Total number of orders** |  | 965,532 | 100% | 553,036 | 100% |  | 388,882 | 100% | 217,105 | 100% |
| **Monthly average number of orders** |  | 68,967 |  | 61,448 |  |  | 27,277 |  | 24,123 | 100% |
|  |  |  |  |  |  |  |  |  |  |  |
|  |  |  |  |  |  |  |  |  |  |  |
| **Race/Ethnicity** |  |  |  |  |  |  |  |  |  |  |
| White |  | 647,846 | 67.1% | 373,371 | 67.5% |  | 228,801 | 58.8% | 134,617 | 62.0% |
| Asian |  | 26,498 | 2.7% | 13,963 | 2.5% |  | 7,482 | 1.9% | 3,709 | 1.7% |
| Black or African American |  | 65,320 | 6.8% | 42,126 | 7.6% |  | 21,547 | 5.5% | 13,127 | 6.0% |
| Hispanic or Latino |  | 72,685 | 7.5% | 41,912 | 7.6% |  | 20,947 | 5.4% | 12,232 | 5.6% |
| Other |  | 15,722 | 1.6% | 9,999 | 1.8% |  | 4,436 | 1.1% | 2,033 | 0.9% |
| Unknown |  | 137,461 | 14.2% | 71,665 | 13.0% |  | 105,669 | 27.2% | 51,387 | 23.7% |
|  |  |  |  |  |  |  |  |  |  |  |

SOURCE: Authors’ analysis of Veradigm ambulatory care electronic health record data from the COVID-19 Research Database.
